# Acceptance and perceived barriers of implementing a guideline for managing low back in general practice

**DOI:** 10.1186/1748-5908-3-7

**Published:** 2008-02-07

**Authors:** Jean-François Chenot, Martin Scherer, Annette Becker, Norbert Donner-Banzhoff, Erika Baum, Corinna Leonhardt, Stefan Keller, Michael Pfingsten, Jan Hildebrandt, Heinz-Dieter Basler, Michael M Kochen

**Affiliations:** 1 Dpt. of General Practice, University of Göttingen, Humboldtallee 38, 37073 Goettingen, Germany; 2 Dpt. of General Practice, Preventive and Rehabilitation Medicine, University of Marburg, Robert-Koch-Str. 5, 35037 Marburg, Germany; 3 Institute for Medical Psychology, University of Marburg, Bunsenstr. 3, 35037 Marburg, Germany; 4 Dpt. of Public Health Sciences, University of Hawaii at Manoa, 1960 East-West Rd., Honolulu, HI 96822, USA; 5 Dpt. of Anesthesiology, Pain Clinic, University of Göttingen, Robert-Koch-Str. 40, 37075 Göttingen, Germany

## Abstract

**Background:**

Implementation of guidelines in clinical practice is difficult. In 2003, the German College of General Practitioners and Family Physicians (DEGAM) released an evidence-based guideline for the management of low back pain (LBP) in primary care. The objective of this study is to explore the acceptance of guideline content and perceived barriers to implementation.

**Methods:**

Seventy-two general practitioners (GPs) participating in quality circles within the framework of an educational intervention study for guideline implementation evaluated the LBP-guideline and its practicability with a standardised questionnaire. In addition, statements of group discussions were recorded using the metaplan technique and were incorporated in the discussion.

**Results:**

Most GPs agree with the guideline content but believe that guideline stipulations are not congruent with patient wishes. Non-adherence to the guideline and contradictory information for patients by other professionals (*e.g*., GPs, orthopaedic surgeons, physiotherapists) are important barriers to guideline adherence. Almost half of the GPs have no access to recommended multimodal pain programs for patients with chronic LBP.

**Conclusion:**

Promoting adherence to the LBP guideline requires more than enhancing knowledge about evidence-based management of LBP. Public education and an interdisciplinary consensus are important requirements for successful guideline implementation into daily practice. Guideline recommendations need to be adapted to the infrastructure of the health care system.

**Trial registration:**

BMBF Grant Nr. 01EM0113. FORIS (database for research projects in social science) Reg #: 20040116 [25].

## Introduction

Low back pain (LBP) is a major medical, social, and economic problem worldwide. Variations in care for this mostly self-limiting condition lead to discrepancies in health care costs without noticeable impact on outcome, such as days in pain or days of sick leave [1]. Recently, several national and European guidelines have been released with the goal of promoting evidence-based care for LBP to direct health care resources and improve quality of care [2].

In 2003, the German College of General Practitioners and Family Physicians (DEGAM) released an evidence-based guideline to improve the management of LBP in general practice [3]. The recommendations of the guideline are concordant with those made by most international guidelines, with some minor adaptations to the national health care system.

Core recommendations are a triage system to identify patients with complicated back pain (red flags) or high risk of chronic back pain (yellow flags), a stepwise diagnostic and therapeutic approach and encouragement of physical activity.

While quality guidelines are becoming increasingly available, implementation of their recommendations remains a daunting task [4,5]. Several randomized controlled trials on the implementation of LBP guidelines showed insignificant or only minimal impact on the management of LBP [6,7]. Guideline implementation in Germany for LBP is further complicated by an unstructured health care system where patients have direct access to ambulatory specialist care without needing a referral from a general practitioner (GP). Therefore GPs compete directly with orthopaedic surgeons and other specialists for the care for patients with LBP.

A European working group in 2002 concluded that implementation strategies should be based on the present knowledge of potentially effective interventions, and should include considerations of available resources for, and potential barriers to, implementation [8]. The aim of this article is to explore the acceptance of guideline recommendations and presumed barriers to guideline adherence in a sample of GPs who participated in quality circles (QCs) within the framework of a randomized controlled trial to implement the DEGAM LBP guideline.

## Methods

This was an educational intervention within a three-armed randomized controlled trial. The primary goal was to assess the impact on patients' outcomes and guideline adherence [9]. Here, we report the results of an evaluation questionnaire and the group discussions of the GPs who participated in the intervention arms.

### General practitioners

We contacted 818 general practices (883 GPs) surrounding both study centers. Addresses were obtained from local health authorities. The areas encompass two medium-sized university cities and surrounding small towns and rural areas, thus being representative for most parts of Germany except for large cities. GPs and practice nurses had to agree to participate in the educational intervention, in case they would be randomized into one of the intervention arms. Fifty percent did not respond, and 34% declined mainly because practice nurses refused to participate. From the 118 (126 GPs) practices who agreed to participate, 74 (80 GPs) were assigned to the intervention arms. GPs received 200 euros for participation in the study.

The educational intervention took place in temporary interactive group sessions organised like QCs for GPs (in both intervention arms), and in the training of practice nurses to give motivational counselling to promote physical activity (in one of the intervention arms). Ethical approval was obtained for both study sites. We conducted eight QCs, four in each region. The number of participants ranged from seven to 14.

QCs, also called peer review groups, are popular in Germany and the Netherlands for continuous medical education [10]. QCs may be described as small groups of physicians (or interdisciplinary groups with other health professionals), based on voluntary participation and concerned with activities aimed at assessing and continuously improving the quality of patient care. Therefore, QCs might be a valuable venue for promoting guideline implementation.

All GPs received a long and short version of the guideline and a set of patient leaflets by mail. Eighty GPs from 74 practices attended at least two of the three QCs (groups of 10–14 GPs) each lasting about two hours during a period of two months. The first session of the QCs focussed on acute LBP. It included interactive case presentations and a short course in physical examination. The second session focused on chronic LBP and patient counselling. The last session was dedicated mainly to the discussion of strategies and barriers to the implementation of the guideline recommendations in practice, after GPs had recruited the first patients with LBP into the trial. To facilitate group discussions, we used the metaplan technique [11]. GPs wrote down comments on cards which were grouped according to themes on a board.

### Data collection and analysis

The educational intervention and the questionnaire were tested in a small pilot study. The feedback from participating GPs influenced the development of the questionnaire. GPs participating in the QCs were asked to anonymously fill out the self-developed questionnaire at the end of the last session in order to assess their agreement with the guideline contents and their confidence about being able to put guideline recommendations into practice. Answers to the questions followed a 4-item Likert format (strongly agree, mostly agree, disagree, strongly disagree).

There was no verbatim transcription of the group discussions. The cards with GPs' comments were the basis for the protocols of the third sessions. Similar comments were summarised and grouped according to main themes. Though many topics were mentioned repeatedly, we did not attempt to quantify how frequently they were mentioned. We only show comments made in at least two different sessions.

## Results

The average age of the 80 participating GPs was 47.9 years (SD ± 6.4) (national average 50.4 years), 46% were female (national average 36%) and they were on average 12 years in practice (SD ± 6.5). Age, gender, and years in practice of our sample are not meaningfully different from the national average [12].

A total of 72 questionnaires (90% of the participating GPs) were returned. Overall, GPs endorsed the guideline in general, as well as the assumptions and recommendations. Only a minority (1 to 3%) disagreed with the guideline (Table 1). While half (56%) of the GPs said the guideline changed their practice of managing LBP, 83% claimed that they already treated patients according to the guideline. Twenty-one percent feared they could lose patients if they adhered to the guideline. Aproximately one-half (54%) of GPs assumed that patients want to have explanations on pathophysiology, and expect extensive diagnostic (45%) and therapeutic (64%) interventions (Table 2).

**Table 1:** Evaluation of the guideline (in %, n = 72).

	Strongly agree	Mostly agree	Disagree	Strongly disagree
The guideline is suitable for daily practice	65	35	∅	∅
The guideline increases my confidence in managing low back pain.	53	44	3	∅
I will lose patients by adhering to the guideline	4	17	47	32
I agree with the information provided with the patient leaflet.	62	37	1	∅
The guideline should be disseminated.	86	14	∅	∅
I have been treating low back pain according to the guideline previously.	39	54	7	∅
The guideline has changed my management of low back pain.	13	43	34	10
Triaging patient with low back pain after history taking and physical exam in uncomplicated, radicular and complicated back pain instead of making an anatomical diagnosis is reasonable	92	5	3	∅
The majority of patients in my practice have uncomplicated back pain.	79	17	3	1
The „yellow flags" are useful to recognize patients at risk for chronic back pain.	54	45	1	∅
To postpone imaging for the first 4–6 weeks is reasonable.	72	27	1	∅
The therapeutic options suggested for acute back pain are helpful.	56	43	1	∅
The therapeutic options suggested for chronic back pain are helpful.	47	53	∅	∅

**Table 2:** Presumed patient expectations (in %, n = 72).

	Strongly agree	Mostly agree	Disagree	Strongly disagree
My patient expect me to clarify the cause of their LBP, otherwise if I postpone diagnostic tests beyond physical examination, I might lose patients.	12	42	42	4
Patient expect extensive diagnostic interventions otherwise the change the physician.	6	39	43	12
Patients expect injection, massage prescriptions or other "new therapies".	13	51	31	5
If I meet patient's expectations in one point (e.g. imaging, injection), I facilitate the promotion of physical activity.	18	49	30	3

While the majority of GPs was satisfied with the cooperation with physiotherapists (75%) and neurologist (68%), cooperation with ambulatory orthopaedic surgery was rated favourably only by 39% (Table 3). More than half of the GPs had no local access to multimodal pain programs for patients with chronic LBP.

**Table 3:** Cooperation with specialist and local infrastructure (in %, n = 72).

	Strongly agree	Mostly agree	Disagree	Strongly disagree
Cooperation with orthopaedic surgeons is good and facilitates guideline adherence.	7	32	42	19∅
Cooperation with neurologist is good and facilitates guideline adherence.	15	53	25	7
Cooperation with physiotherapists is good	25	50	19	6
Cooperation with radiologists is good and facilitates guideline adherence.	22	26	29	13
I have access to multimodal rehabilitation for patients chronic LBP.	21	24	25	30

Main topics from the group discussions extracted from the protocols and grouped in topics are shown in Table 4. They concern the guideline in general and discuss some diagnostic and therapeutic procedures as well as cooperation with other health care providers.

**Table 4:** Comments subtracted using the metaplan technique from group discussions of GPs.

**Topic**	**Comments of GPs**
Guideline in general	■ Patients need to be taken serious ■ Guideline downplays patients' pain
Communication	■ Difficulties conveying the non-biomechanic diagnosis ■ Mentioning the guideline approved by university increases credibility ■ Difficulties "selling" psychotherapy for LBP
Physical activity	■ Is easier to promote in younger people ■ Is mainly attractive for women ■ It is hard to motivate elder man ■ It is hard to motivate and give reasons for physical activity to physically hard working patients
Physiotherapy	■ Patient are highly satisfied with physical therapy ■ Knowledge deficits about what physical therapist can do ■ Suspicion that PT change prescription for physical therapy into massage
Imaging	■ General agreement on its low impact on patient care and therapeutic decisions ■ Patients want imaging ■ Increases prestige of the condition ■ Refusal of imaging could be perceived as cost-saving measure ■ Postponing imaging requires more counselling time
Cooperation with orthopaedic surgeons	■ Orthopaedic surgeons are (ab)used to get rid of difficult patients. ■ Fear of being blamed of missing something albeit not important ■ Troubles with access for patients with suspicion of serious complication or severe pain ■ Routine imaging and routine prescription of physiotherapy by orthopaedic surgeons make GPs appear as "poor man's choice"
Injections	■ Injections are popular particular among elder patients ■ Replacement of injections with non-steroidals by injections of local anaesthetics
Patient education	■ There should be public education on the radio and on tv about the ineffectiveness of bed rest, imaging etc.

## Discussion

### Main findings

More than 90% of the GPs in our study agreed with the core assumptions and recommendations made by the guideline and believed it is helpful. However, guideline adherence in daily practice is considered problematic. The main barriers were fear of not meeting patient expectations, unsatisfactory cooperation with specialists, and a lack of access to multimodal programs.

There is a discrepancy between the claim of having treated patients previously in accordance with the guideline recommendations, and the statement that the guideline has changed their management of LBP.

### Strengths and limitations

We have a large sample of GPs who, with regarding age and gender distribution, are not meaningfully different from the national average. However, our sample may not be representative since GPs with general objections to guidelines might have been less likely to participate in this implementation intervention study. Answers to the questionnaires might partly be due to social desirability and selection bias, and not necessarily reflect real behaviour. Agreement with guideline recommendations of GPs in general might be lower. The main purpose of the QCs was educational, and we did not perform an in-depth qualitative study.

### Meaning of the results and comparison with other studies

Agreement is a basic but not sufficient precondition for guideline implementation [13]. In accordance with Schers *et al*., perceived patient preferences were seen as an important obstacle for adhering to the guideline in our study [14]. Given the excellent short-term prognosis of LBP, the epidemiological model on which most LBP-guidelines are based purposefully leaves most cases of back pain etiologically unexplained. Therefore, the guideline suggests postponing extensive diagnostic evaluation in the absence of warning signs for complicated LBP. This conflicts with the traditional biomechanical model postulating a specific anatomical cause [15]. During group discussions, GPs admitted difficulties in conveying the epidemiologic concept of unspecific LBP to the patient, which was also found by Miller *et al*. [16]. Some GPs suggested the use of anatomical models for patient counselling, while others discouraged the use of models as counterproductive. Although most GPs agree with the guideline that intensive diagnostic procedures can be postponed, a large proportion (45%) assumed that patients expect diagnostic interventions. In group discussions, it became clear that GPs feared that postponing diagnostic and therapeutic interventions might be perceived as a cost-cutting measure. They were also concerned that patients might feel that their pain was not being taken serious, and that their condition was being downplayed.

Since it has been shown that most patients with LBP in primary care seek reassurance and advice, this assumption about patients' expectations might be wrong [17,18]. Since these assumptions undermine the guideline recommendations to a certain extent, GPs probably provide themselves a welcome argument to go on with their traditional management of LBP.

The GPs in our study mentioned that they would continue to manage older patients the traditional way (injection therapies, bed rest) because they were used to it, but manage younger patients according to the guideline (oral analgesics, activity as tolerated). This is also in accordance with Schers *et al*. [19]. This dichotomy may explain in part the contradiction between GPs' self-reported already high level of guideline concordant patient management and the perceived high impact of the guideline implementation on their patient management.

Patients in Germany are not enlisted with a fixed GP, and have almost unrestricted access to all doctors and ambulatory specialty care. They do not need a referral to see a specialist, *e.g*., an orthopaedic surgeon. This opens the door for 'doctor shopping'. Therefore, colleagues giving into assumed or real patient expectations which are not guideline concordant were regarded as a problem. They increase the pressure to fulfil patient preferences for inappropriate diagnostic or therapeutic procedures. This is particularly a problem if patients receive contradictory information about the aetiology, diagnostic procedures, treatment, and prognosis of LBP by other health care providers. A typical described situation was the orthopaedic surgeons or another GP ordering inappropriate imaging. The frequent finding of a small disc prolapse or degenerative changes in patients with no neurological symptoms discredits the primary care providers' diagnostic abilities by providing a plausible, albeit medically irrelevant, explanation for LBP. It has been shown that although imaging increases patient satisfaction, it negatively affects the outcome, like pain [20,21]. Information and advice from health care providers have an important impact on patients' perception of the usefulness of imaging [22]. This conflict is reflected by the relatively low satisfaction of GPs with orthopaedic surgeons. Interdisciplinary agreement on management principles of LBP has been recognized as an important factor for successful guideline implementation [23]. Similar problems arose with topics like inappropriate injections of non-steroidal anti-inflammatory drugs or steroids, and inadequate early prescriptions of physiotherapy.

Patient frequently receive contradictory information from different health care providers. Therfore GPs expressed a desire that all health care providers give more congruent and consistent patient information on LBP. Frequently they suggested public education might help to achieve this goal. The effectiveness of a public education program on patients' and GPs' back pain beliefs has been shown in an award winning Australian study [24].

Less than half of the GPs in our study have local access to multimodal pain programs for chronic LBP, as suggested as appropriate by the guideline. Thus, structural barriers like lack of access to recommended treatment options prevent guideline-concordant patient management. The guideline summarises scientific evidence for diagnostic and therapeutic procedures but does not sufficiently reflect the structures of the health care system.

## Conclusion

Presumed patient expectations that are not concordant with guideline recommendations and deficits in cooperation with specialist care are the main barriers to guideline implementation. A common message and congruent information for patients with LBP is important.

We believe that this goal can be achieved when there is a consensus among all involved health professionals on how to manage LBP. In addition, public education, including demythologizing some common beliefs on LBP, is necessary. Guidelines should be adapted to the existing health care structures to facilitate guideline concordant patient care, and in turn health care systems should provide structures that facilitate guideline adherence.

## Competing interests

The author(s) declare that they have no competing interests.

## Authors' contributions

All authors contributed to study design. QCs were lead by AB, EB, HDB, JFC, JH, MP and NDB. All authors contributed to manuscript drafting and revision and approved the final manuscript.
